# Escherichia fergusonii, an Underrated Repository for Antimicrobial Resistance in Food Animals

**DOI:** 10.1128/spectrum.01617-21

**Published:** 2022-02-09

**Authors:** Biao Tang, Jiang Chang, Yifei Chen, Jiahui Lin, Xingning Xiao, Xiaodong Xia, Jun Lin, Hua Yang, Guoping Zhao

**Affiliations:** a State Key Laboratory for Managing Biotic and Chemical Threats to the Quality and Safety of Agro-products & Institute of Agro-product Safety and Nutrition, Zhejiang Academy of Agricultural Sciences, Hangzhou, Zhejiang, China; b School of Agriculture and Biology, Shanghai Jiao Tong University, Shanghai, China; c College of Food Science and Engineering, Northwest Agriculture and Forestry University, Yangling, Shaanxi, China; d Department of Animal Science, The University of Tennessee, Knoxville, Tennessee, USA; e CAS Key Laboratory of Synthetic Biology, Institute of Plant Physiology and Ecology, Shanghai Institutes for Biological Sciences, Chinese Academy of Sciences, Shanghai, China; University of Pittsburgh School of Medicine

**Keywords:** *Escherichia fergusonii*, food animal, antimicrobial resistance, *mcr-1*

## Abstract

A total of 1,400 samples of food animals (pigs, chickens, and ducks) were collected between July and September 2019 in China to uncover the prevalence of *E. fergusonii* and its potential role in the evolution of antimicrobial resistance (AMR). An isolation of *E. fergusonii* was performed and pulsed-field gel electrophoresis (PFGE) was used to uncover the genetic relationship. The AMR of *E. fergusonii* isolates was comprehensively characterized using broth microdilution-based antimicrobial susceptibility testing, S1-PFGE, southern hybridization, whole-genome sequencing, and in-depth bioinformatics analysis. As a result, a total of 133 *E. fergusonii* isolates were obtained. These isolates could be grouped into 41 PFGE subclades, suggesting a diverse genetic relationship. The resistance phenotypes of sulfafurazole (97.74%) and tetracycline (94.74%) were the most frequently found. Of the *E. fergusonii* isolates, 51.88% were extended spectrum beta-lactamase (ESBL)-positive. Forty-three different AMR genes were revealed based on 25 genome sequences harboring *mcr-1.* Briefly, *aph*(6)*-Id*, *aph*(3′′)*-Ib* and *tet*(A) genes were the most frequently observed, with the highest rate being 76.00% (19/25). Three *mcr-1*-harboring plasmids were identified after Nanopore sequencing, including pTB31P1 (IncHI2-IncHI2A, 184,652 bp), pTB44P3 (IncI2, 62,882 bp), and pTB91P1 (IncHI2-IncHI2A, 255,882 bp). Additionally, 25 *E. fergusonii* isolates harboring *mcr-1* were clustered together with other *E. fergusonii* isolates from different regions and sources available in GenBank, suggesting a possible random process of *mcr-1* transmission in *E. fergusonii*. In conclusion, *E. fergusonii* is widespread in food animals in China and might be an important reservoir of AMR genes, especially *mcr-1,* and facilitate the evolution of AMR.

**IMPORTANCE** *E. fergusonii*, a member of the genus Escherichia, has been reported to transmit via the food chain and cause diseases in humans. However, the prevalence of multidrug-resistant *E. fergusonii*, especially *mcr-1*-positive *E. fergusonii* isolates, has rarely been reported. Here, we collected 1,400 samples from food animals in three provinces of China and obtained 133 *E. fergusonii* isolates (9.5%). We found that the prevalence of *E. fergusonii* isolates was diverse, with high levels of antimicrobial resistance. Among them, 18.8% *E. fergusonii* isolates carried the colistin resistance gene *mcr-1*. Thus, *E. fergusonii* may facilitate the evolution of colistin resistance as a reservoir of *mcr-1.* As far as we know, the prevalence and AMR of *E. fergusonii* in the food animals in this study was first reported in China. These findings increase our understanding of the role of *E. fergusonii* in public health and the evolution of antibiotic resistance.

## INTRODUCTION

Escherichia is an important genus of *Enterobacteriaceae* bacteria. To date, there are nine species in the Escherichia genus, including Escherichia coli, Escherichia fergusonii, Escherichia albertii, Escherichia blattae, Escherichia hermanii, Escherichia vulneris, Escherichia adecarboxylata, Escherichia marmotae, and Escherichia ruysiae (1) (https://lpsn.dsmz.de/genus/escherichiaescherichia). *E. fergusonii*, as a new species distinguished from E. coli, was first detected in a clinical blood sample in 1985 (2). Subsequently, *E. fergusonii* has been isolated from other clinical cases, including wound infections, urinary tract infections, bacteremia, and diarrhea, indicating its ability to cause diseases and infections (3–5). In addition, *E. fergusonii* was also found to be prevalent in foods (6, 7) and food animals (8–10), indicating that it is potentially widespread in the food chain. Based on the above characteristics, *E. fergusonii* could pose a potential risk to food safety and public health.

It has been reported that multidrug-resistant *E. fergusonii* has been isolated from both clinical cases and food animals, which suggests that antimicrobial resistance (AMR) might occur in this species (10–12). In addition, it was reported that diverse AMR genes were detected in *E. fergusonii,* including some notorious genes such as extended spectrum beta-lactamases (ESBLs) (12), further indicating that *E. fergusonii* might be an important reservoir of AMR genes. Therefore, it was speculated that *E. fergusonii* might play an important role in AMR transmission. However, the characteristics of antibiotic resistance in *E. fergusonii* have not been comprehensively studied.

Colistin, a peptide antibiotic, was one of the antibiotics of last resort for the treatment of multidrug-resistant, especially carbapenem-resistant, Gram-negative pathogen infections (13, 14). However, there has been an increasing trend of *Enterobacteriaceae* resistance to colistin in recent years (15–17). In 2016, *mcr-1*, a plasmid-borne resistance gene, was identified as the main factor contributing to the widespread colistin resistance which poses a serious risk to patient health (13, 18). An *mcr-1*-positive *E. fergusonii* was first detected in 2018 in Guangdong, China (14). Subsequently, an *E. fergusonii* plasmid with a complete sequence that harbored both *mcr-1* and ESBLs was identified from chicken feces in 2019 in Zhejiang, China, suggesting that *E. fergusonii* might play an important role in the transmission of *mcr-1* and pose a serious threat to clinical infection treatment (19). Therefore, a comprehensive examination of the characteristics of *mcr-1*-positive *E. fergusonii* is also highly warranted.

In this study, a comprehensive study of the prevalence and characterization of *E. fergusonii* in food animals was performed. The PFGE profiles and AMR of *E. fergusonii* isolates were uncovered and discussed. In addition, novel insight into the features and routes of phylogenetic relationship of *mcr-1*-positive *E. fergusonii* and *mcr-1*-harboring plasmids was obtained. These results highlight the serious threat *E. fergusonii* poses to public health and greatly improve our understanding of its role in the transmission and development of antibiotic resistance.

## RESULTS

### The prevalence and PFGE profile of Escherichia fergusonii.

**FIG 1:**
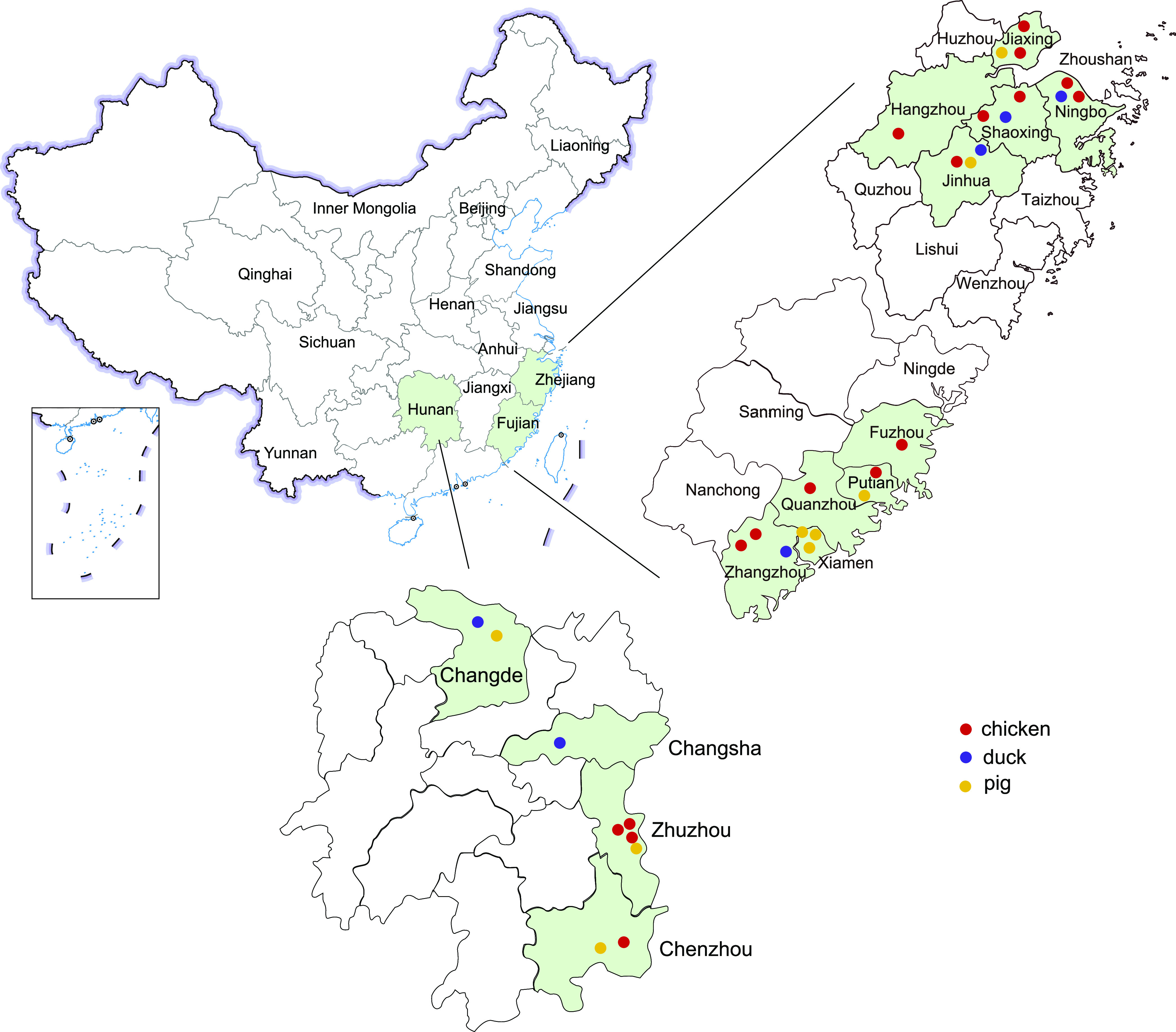
Map of sampling areas in Zhejiang, Fujian, and Hunan provinces, China. The sampling sources of the chicken, duck, and pig are denoted with red, purple, and yellow circles, respectively. Zhejiang, Fujian, and Hunan provinces in this study are shaded in light green.

In total, 133 *E. fergusonii* isolates were isolated from 1,400 samples at 23 different locations (Fig. 1), with a total isolation rate of 9.50% (Table S1 in the supplemental material). Among these isolates, 84 isolates were isolated from chicken-source samples, with the highest isolation rate being 11.67%. The isolation rates of duck and pig samples were 7.41% (20/270) and 7.07% (29/410), respectively. In addition, the distribution of isolation rates in different locations ranged from 0% to 36%. *E. fergusonii* was not detected in 9 sampling sites.

**FIG 2:**
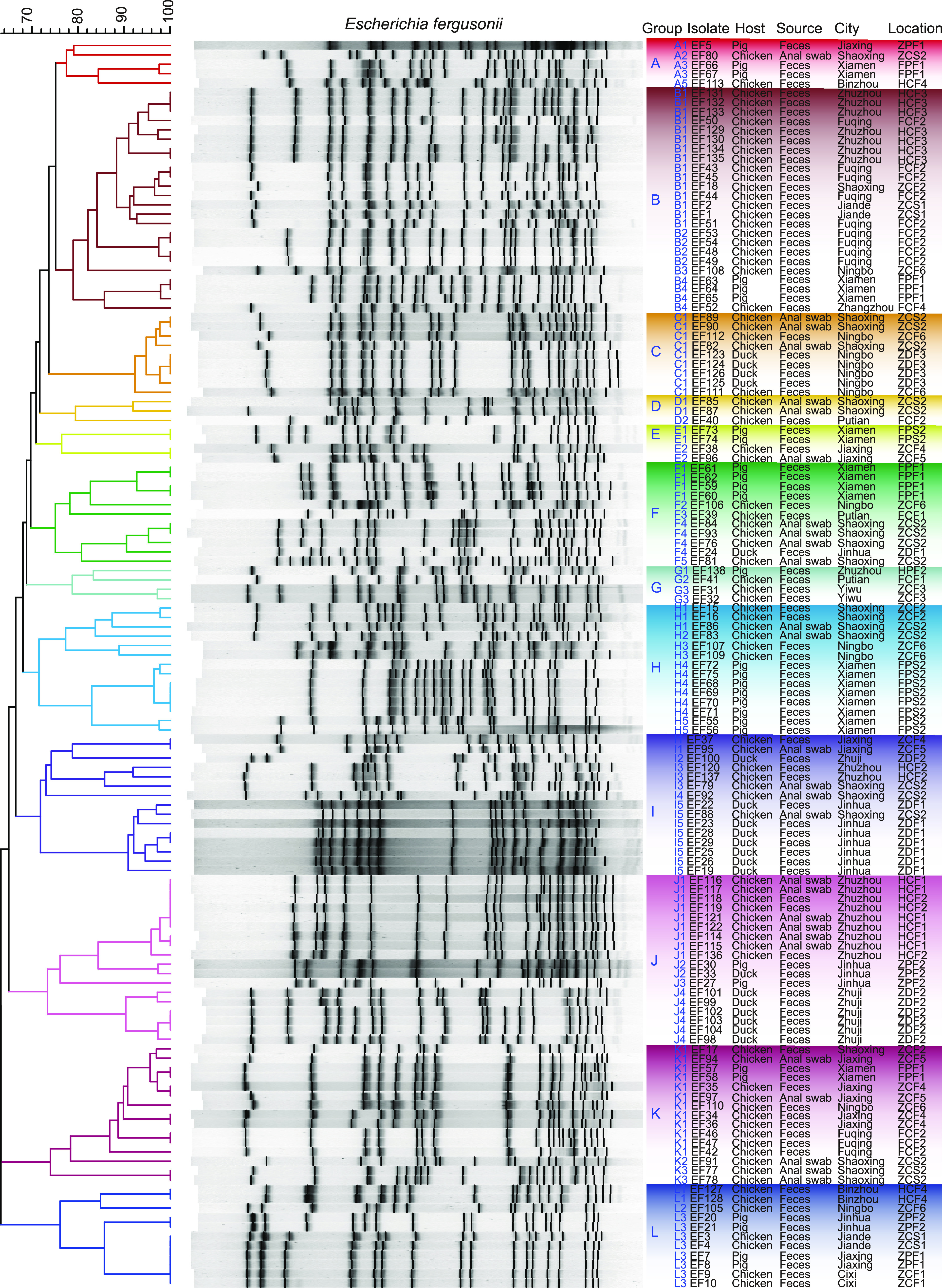
PFGE patterns of 133 *E. fergusonii* isolates. PFGE pattern similarity was assessed by cluster analysis and band-matching applications of BioNumerics software (Applied Maths, Sint-Martens-Latem, Belgium). PFGE groups of *E. fergusonii* were defined at similarity cutoff values of 70% and 85%. Detail information for different locations is shown in Table S1 in the supplemental material.

To identify the genetic relatedness of *E. fergusonii* isolates recovered from different animal sources and locations, all 133 isolates were subtyped using pulsed-gel electrophoresis (PFGE). As shown in Fig. 2, at similarity cutoff values of 70% and 85%, DNA profiles of the 133 isolates were grouped into 12 PFGE types and 41 subclades, which included 93 PFGE fingerprints with a similarity of 63.1% ∼ 100.0%. Among the 12 PFGE types, group B (24/133, 18.05%) was the most common PFGE type, followed by group J (18/133, 13.53%).

The PFGE types of isolates from different animal sources showed different features, and there were many PFGE types in isolates from the same animal source. In detail, there were 30 (30/41, 73.17%) PFGE subclades in chicken-source isolates, and the prevalent subclades were B1, K1, and J1. There were 24 PFGE subclades only found in chicken-source isolates. In addition, there were 12 subclades specifically identified in pig-source isolates and 6 subclades identified in duck-source isolates. It was found that the isolates from different animal sources could share the same PFGE subclade. For example, each of the subclades B4, C1, F4, I5, J2, K1, and L3 included isolates from two different animal sources (Fig. 2). However, there were no subclade present in three different animal source isolates in this study.

Multiple PFGE subclades also could be identified in the isolates from a single region. Among these regions, the number of PFGE subclades in Shaoxing was the greatest (14/41, 34.15%), followed by Xiamen (7/41, 17.07%). In addition, it was found that the same PFGE subclade could be prevalent in different regions. For example, K1 was identified in different isolates from Shaoxing, Jiaxing, Fuqing, Xiamen, and Ningbo, and B1 was identified in isolates from Jiande, Shaoxing, Fuqing, and Zhuzhou. Moreover, multiple PFGE subclades could be identified in the isolates from single location (Fig. 2). For example, A2, C1, D1, F4, H1, H2, I3, I4, K2, and K3 were identified in different isolates from farm ZCS2, suggesting that there might be diverse transmission sources in a single farm. Meanwhile, it was also found that different *E. fergusonii* isolates from a single location had the same PFGE subclade. For example, strains EF68 to EF71, belonging to H4, were isolated from FPS2, and strains EF131 to EF133, belonging to B1, were isolated from HCF3. This phenomenon suggested that clone spread may be occurring. Thus, the PFGE profile results showed that there was a complex transmission mode of *E. fergusonii* isolates in a single sampling location.

### The phenotype of antimicrobial resistance.

**FIG 3:**
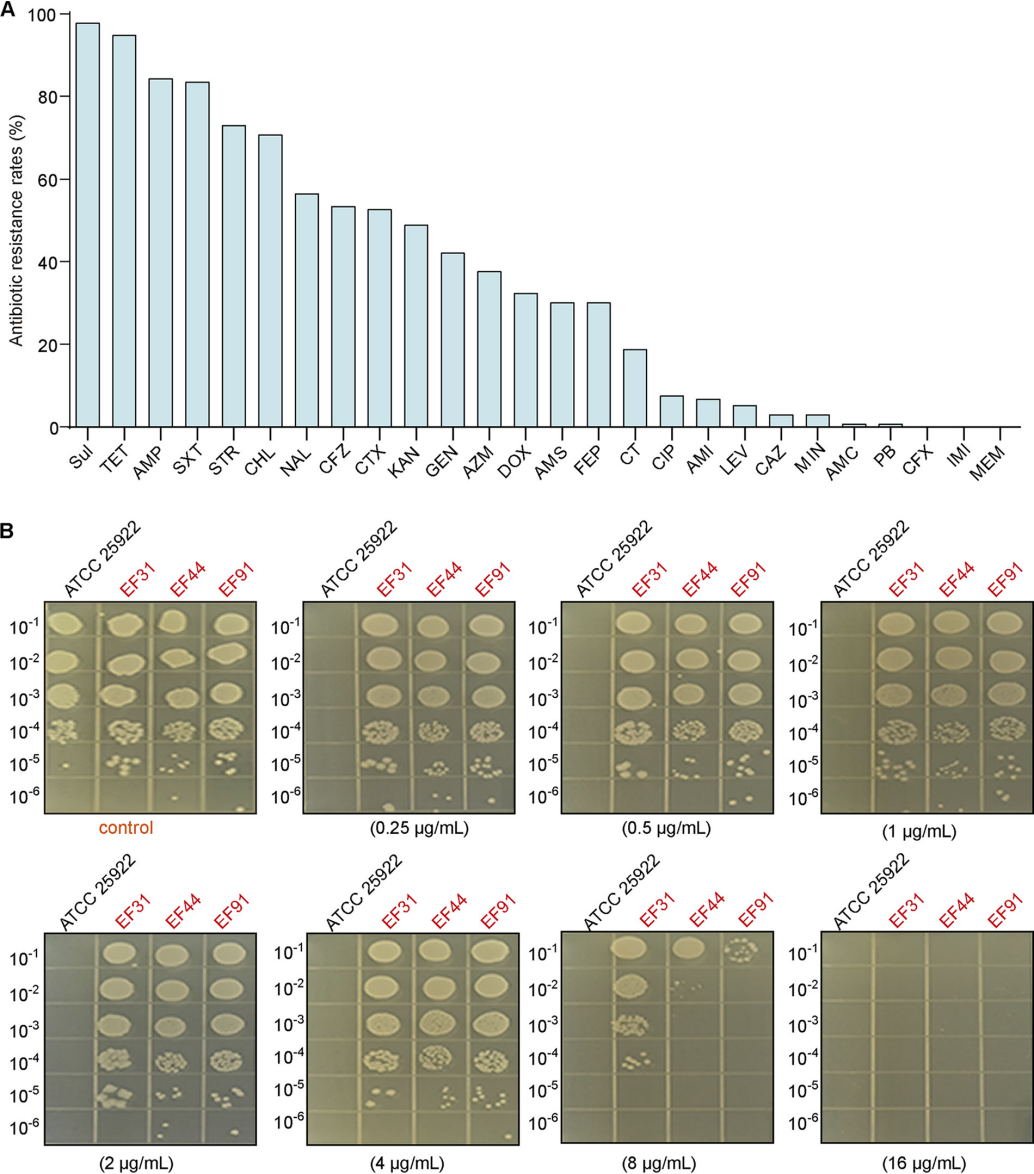
Antibiotic resistance phenotypes of *E. fergusonii* isolates. (A) Resistance rates of *E. fergusonii* isolates to 26 antibiotics. (B) The insusceptibility of strains EF31, EF44 and EF91 under colistin pressure was determined by the agar dilution method. E. coli ATCC 25922 served as a quality control strain.

The MICs for all antimicrobial agents (Table S2**)** of 133 strains were obtained by microbroth dilution method (Table S3), and the MIC distributions are shown in Fig. S1 in the supplemental material. Among 133 *E. fergusonii* isolates, the highest level of resistance was observed for sulfafurazole (SUL), with 97.74% isolates, followed by tetracycline (TET) (94.74%), ampicillin (AMP) (84.21%), and sulfamethoxazole (SXT) (83.46%) (Fig. 3A). 18.80% of isolates were resistant to colistin (CT), while none of the *E. fergusonii* isolates showed resistance to meropenem (MEM) or imipenem (IMI). In addition, 51.88% of *E. fergusonii* isolates were ESBL-positive. All 133 *E. fergusonii* isolates were resistant to at least one antimicrobial agent, while 128 isolates (96.24%) showed resistance to four or more antimicrobial agents. In particular, one isolate was found to be resistant to 20 antimicrobials (Fig. S2).

### Characterization of the genome features of colistin-resistant *E. fergusonii* isolates.

Whole-genome sequencing was performed on all 25 colistin-resistant *E. fergusonii* isolates. As shown in Fig. S3, there were 11 plasmid replicons predicted across all isolates, with an average of 5.2 plasmid replicons in each isolate. The IncI2 and IncX1 plasmids were both predicted in 22 of the 25 isolates (88.00%) and were the most prevalent plasmid types in colistin-resistant *E. fergusonii*.

**FIG 4:**
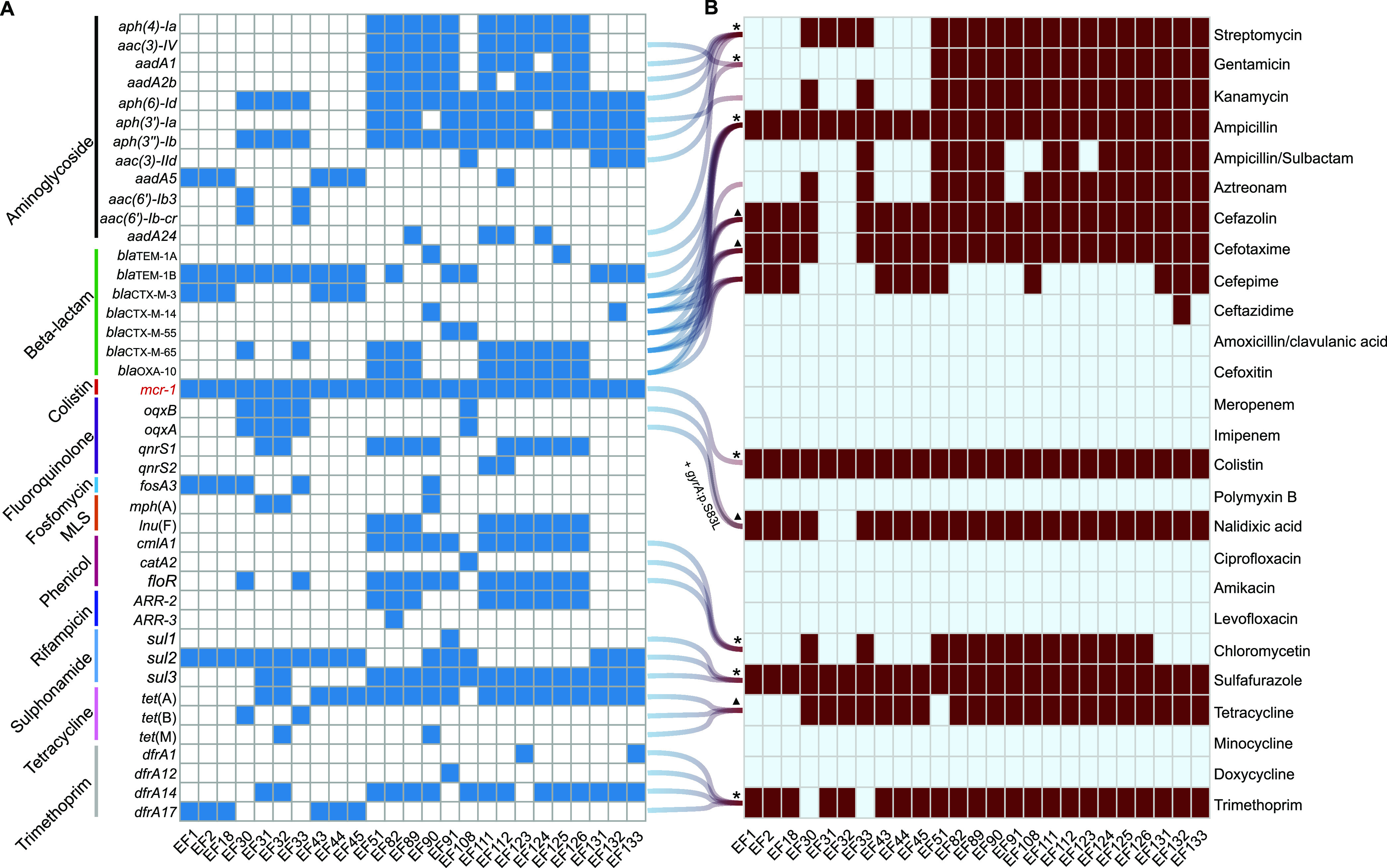
Correlation of AMR genes and phenotypic susceptibility testing. (A) Acquired AMR genes in colistin-resistant *E. fergusonii* isolates. Blue represents presence of the gene and white represents absence of the gene. (B) AMR profiles of 25 *E. fergusonii* strains. Brownish red indicates resistance and light blue indicates sensitivity. Asterisks represent 100% consistency and triangles represent >92% consistency.

There were 43 antibiotic resistance genes detected in all *mcr-1*-positive isolates (Fig. 4), which mainly lead to some resistance phenotypes, including aminoglycoside, beta-lactam, colistin, fluoroquinolone, fosfomycin, macrolides-lincosamides-streptogramin B (MLS), phenicol, rifampicin, sulfonamide, tetracycline, and trimethoprim resistance. All 25 isolates harbored at least seven kinds of AMR genes, and 44.00% (11/25) of isolates harbored at least 15 kinds of AMR genes. In particular, both EF82 and EF112 isolates harbored 20 kinds of AMR genes, which shows serious potential for multidrug resistance. The *mcr-1* gene was detected in all isolates, and *aph*(6)*-Id*, *aph*(3′′)*-Ib* and *tet*(A) genes were the most prevalent, with rates of 76.00% (19/25). In particular, some notorious antibiotic resistance genes also had high coharboring rates with *mcr-1*, such as *bla*_TEM-1B_ (64.00%, 16/25), *floR* (52.00%, 13/25), *bla*_CTX-M-65_ (44.00%, 11/25), *bla*_OXA-10_ (36.00%, 9/25), *bla*_CTX-M-3_ (24.00%, 6/25), *bla*_TEM-1A_ (8.00%, 2/25), *bla*_CTX-M-14_ (8.00%, 2/25), and *bla*_CTX-M-55_ (8.00%, 2/25). In addition, a *gyrA*:p.S83L mutation (leading to quinolone resistance) was detected in 88.00% (22/25) of *mcr-1*-positive isolates (Table S4**)**.

Acquired AMR genes can be 100% consistent with the phenotypic susceptibility, such as streptomycin, gentamicin, ampicillin, colistin, chloromycetin, sulfafurazole, and trimethoprim. In addition, genome sequences can explain 92% of the AMR profiles for cephalosporin, cefotaxime, tetracycline, and nalidixic acid (including *gyrA*:p.S83L mutations) (Fig. 4**)**. All 25 *mcr-1*-positive *E. fergusonii* isolates were multidrug resistant, which might be caused by a variety of acquired AMR genes and chromosomal point mutations.

In addition, a total of 25 *mcr-1*-positive *E. fergusonii* isolates were used to perform S1-PFGE and southern hybridization. As shown in Fig. S4A, all 25 isolates were found to harbor 4 ∼ 6 plasmids, and the size of these plasmids was about 50 ∼ 300 kb in length. All *mcr-1* genes were located on plasmids. The sizes of *mcr-1*-harboring plasmids in strains EF1, EF2, EF18, EF30, EF33, EF43, EF44, EF45, EF51, EF82, EF89, EF90, EF108, EF111, EF112, EF123, EF124, EF125, EF126, EF131, EF132, and EF133 were about ∼60 kb. The sizes of *mcr-1*-harboring plasmids in EF31 and EF32 isolates were about 180.0 kb in length, and the largest plasmid was ∼250 kb in strain EF91. As shown in Fig. S4B, 23 of 25 *mcr-1*-harboring plasmids could successfully transfer to the recipient strain J53, and the conjugation frequency ranged from 10^−4^ to 10^−2^, which suggested that the *mcr-1*-positive plasmids in *E. fergusonii* could be as an important factor for interspecies transmission of colistin resistance.

### Phylogenetic and comparative analysis of *mcr-1*-harboring *E. fergusonii*.

**FIG 5:**
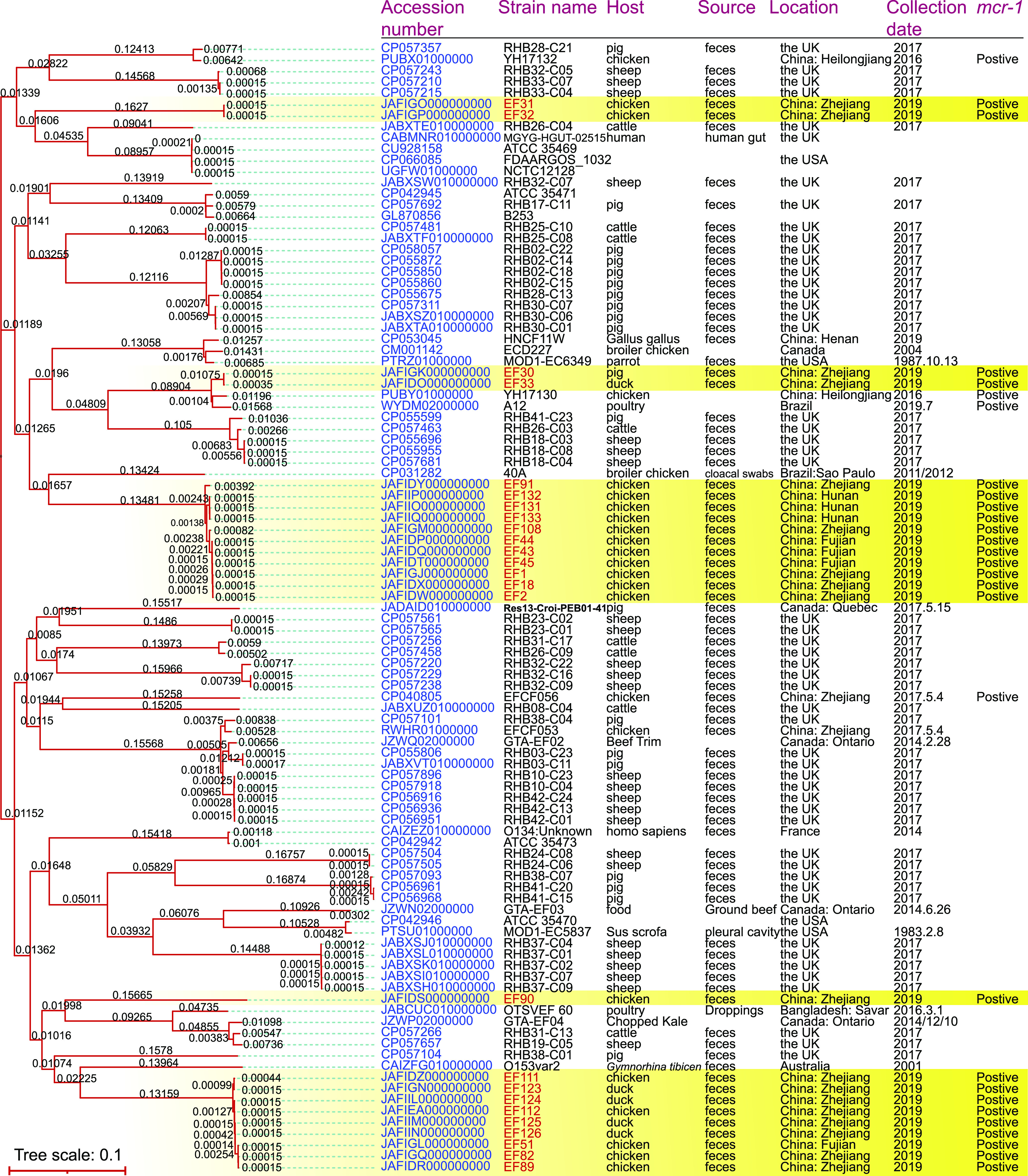
Phylogenetic tree of 101 *E. fergusonii* isolates (76 isolates obtained from GenBank database and 25 isolates in this study) with complete sequences. Yellow background indicates the 25 isolates harboring *mcr-1* in this study. Sequences were aligned for phylogenetic analysis based on the maximum-likelihood method. Blank spaces in the table indicate missing information.

The complete sequences of 25 *mcr-1*-harboring *E. fergusonii* in this study were compared with all complete sequences of *E. fergusonii* available in the GenBank database (up to June 2021), isolated from a variety of sources and countries, and a phylogenetic tree was built based on a single-nucleotide polymorphism (SNP) analysis using the Maximum Likelihood (ML) method. As shown in Fig. 5, some isolates identified from different hosts, sources, locations, and collection dates could be clustered together. At the same time, isolates from the same region could also be in different lineages. In particular, the *E. fergusonii* isolates prevalent in different countries could be clustered in the same lineage, suggesting spread between countries. Although the *E. fergusonii* isolates in this study had a potential phenomenon of clustering together, several isolates showed a closed phylogenetic relationship with isolates from different hosts, sources, locations, and collection dates, such as EF31, EF32, and EF90. Combined with the cluster results of previously reported *mcr-1*-positive isolates WYDM02000000, CP040806, PUBX01000000, and PUBY01000000, and the 25 *mcr-1*-positive isolates in this study, it was found that there is no obvious relationship between *mcr-1* and strain features, suggesting that the *mcr-1* transmission in *E. fergusonii* might be a random process.

Based on the results above, 3 *E. fergusonii* isolates (EF31, EF44, and EF91) with the *mcr-1* gene carried in different-sized plasmids were further chosen for complete genome sequencing. In addition, these strains were selected for survival verification under colistin pressure by the agar dilution method. It was obvious that they grew well at a colistin concentration of less than 4 μg/mL compared with the control (Fig. 3B). Subsequently, *mcr-1*-harboring plasmids were identified in the 3 *E. fergusonii* isolates above, including pTB31P1 (accession no. CP070952) in strain EF31, pTB44P3 (accession no. CP070957) in strain EF44, and pTB91P1 (accession no. CP070874) in strain EF91 (Fig. S5). The plasmid pTB31P1, which is a combination of IncHI2-, IncHI2A-, and IncN-type plasmids, was determined to be 184,652 bp in length and have an average GC content of 46.4%. Except for the *mcr-1* gene, 12 antibiotic resistance genes, such as *bla*_TEM-1B_, *oqxA*, and *oqxB*, were harbored by pTB31P1. The plasmid pTB44P3, which is a IncI2-type plasmid, was determined to be 62,882 bp in length and have an average GC content of 42.9%. This plasmid only harbored the *mcr-1* gene. The plasmid pTB91P1, which is a combination of IncHI2- and IncHI2A-type plasmids, was determined to be 255,882 bp in length and have an average GC content of 46.3%. Except the *mcr-1* gene, 14 AMR genes, such as *floR*, *fosA3*, and *bla*_CTX-M-137_, were harbored by pTB91P1.

An SNP-based phylogenetic analysis was conducted using 124 complete sequences (46 IncHI2-type plasmid sequences and 78 IncI2-type plasmid sequences) of *mcr-1*-harboring plasmids available in GenBank. As shown in Fig. S6 and S7, pTB31P1 was clustered together with CP034788, KX856066, KY990887, MG656414, MG825373, MH459020, MK355502, and MK477611; pTB44P3 was clustered together with KU934208, KX592672, KX772778, KY363994, KY363995, KY405001, KY471312, KY795977, and KY802014; and pTB91P1 was clustered together with CP033224, KU341381, KX084394, KX856065, MK477608, and MK477609. As shown in Fig. S5, the pTB31P1, pTB44P3, and pTB91P1 plasmids shared the same backbone with their similar plasmids. The main difference were some insertion sequences, AMR genes, and type IV secretion system (T4SS). In particular, plasmid pTB31P1 lacked some T4SSs necessary for conjugation, suggesting that its ability to transfer might be impaired. The highly similar plasmids above were isolated from different hosts, including clinical patients, foods, and food animals in different countries, and were harbored by multiple kinds of *Enterobacteriaceae*, including E. coli, Shigella sonnei, Salmonella enterica, and Klebsiella pneumoniae, which demonstrates a diverse and complex transmission mode.

**FIG 6:**
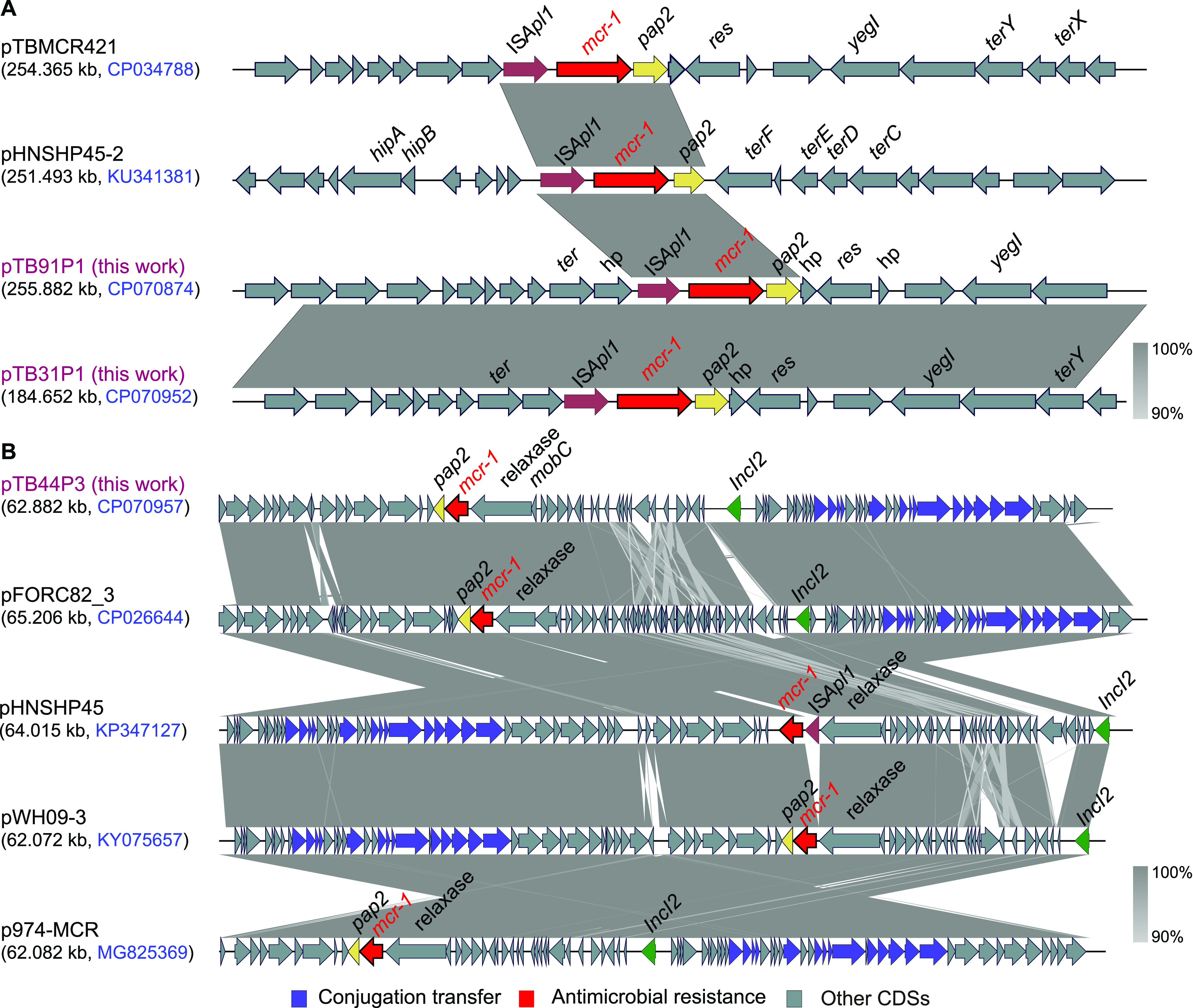
Genetic environment of *mcr-1* in the plasmids pTB31P1, pTB44P3, and pTB91P1. (A) Alignment of pTB31P1 and pTB91P1 with pTBMCR421 and pHNSHP45-2 which harbored a “IS*Apl1*-*mcr-1*-*pap2*” genetic structure. (B) Alignment of pTB44P3 with pFORC82_3, pHNSHP45, pWH09-3, and p974-MCR which harbored a “*mcr-1*-*pap2*” genetic structure.

To uncover the genetic context of *mcr-1* in the different plasmids in this study, a comparative analysis was performed. As shown in Fig. 6, both pTB31P1 and pTB91P1 had a structure of IS*Apl1*-*mcr-1*-*pap2*, which showed a high similarity with the plasmids pHNSHP45-2 and pTBMCR421. In the plasmid pTB44P3, the genetic structure of *mcr-1*-*pap2* was similar to that of plasmids pFORC82_3, pHNSHP45, pWH09-3, and p974-MCR, which lacked an IS*Apl1* upstream of *mcr-1* compared with pTB31P1 and pTB91P1.

## DISCUSSION

It was reported that clinical cases caused by *E. fergusonii* have occurred in many countries (4, 5, 20), posing a serious risk to public health. In particular, *E. fergusonii* could potentially have an important role in antibiotic resistance transmission, and there might be a complex and diverse transmission chain similar to that of E. coli and other *Enterobacteriaceae* strains. It has been reported that food animals play a key role in the transmission of antibiotic resistance genes (13, 21). However, the prevalence of *E. fergusonii* in food animals, and its potential role in the transmission of antibiotic resistance, are poorly understood. In this study, a comprehensive investigation of the prevalence and antibiotic resistance of *E. fergusonii* in food animals was performed. To the best of our knowledge, this is the first report that reveals the features of *E. fergusonii* in food animals and increases our understanding of the diversity and complexity of *E. fergusonii* in China.

From the results, there was a 9.50% isolation rate of *E. fergusonii* in food animals, which suggests that food animals are an important reservoir of *E. fergusonii*. Chicken-source samples had the highest isolation rate, followed by duck-source samples and pig-source samples, which had similar isolation rates. In the past, ducks have often been ignored as a potential reservoir of pathogens; however, there have been increasing reports about the prevalence of pathogens, including multidrug pathogens, from duck sources (22, 23). The results in this study are consistent with previous reports which highlight the important role of ducks in the transmission and development of antimicrobial resistance in pathogens, which could pose a serious threat to food safety and public health. In addition, the *E. fergusonii* isolates in this study had a variety of PFGE profiles, and based on the diversity of PFGE types from different sources and regions, the prevalence of *E. fergusonii* is diverse. This increases the difficulty of controlling *E. fergusonii* and highlights the need for long-term monitoring.

The *E. fergusonii* isolates in this study demonstrated serious antibiotic resistance, especially the colistin-resistant *E. fergusonii* isolates. Plasmids, a type of mobile genetic element, facilitate the development of antibiotic resistance (24). In this study, a variety of Inc-type plasmids harboring many antibiotic resistance genes were identified in the *E. fergusonii* isolates. These plasmids are prevalent in different antibiotic-resistant pathogens (16). To our knowledge, this is the first report that describes the characteristics of plasmids harbored by a range of *E. fergusonii* isolates. Although the database in this study was limited, our results suggest that *E. fergusonii* could be the host of plasmids carrying AMR genes and mediate the development of antibiotic resistance.

Colistin has been banned as a growth-promoting agent in livestock and poultry breeding in China since 2017 (25), and the colistin resistance rate of E. coli of food animal origins decreased to 5% in 2018, compared with 18.1% to 34.0% before 2017 (17). Surprisingly, the isolation rate of *mcr-1*-positive *E. fergusonii* in this study was 18.80%, higher than that of other zoonotic bacteria, such as E. coli (1.55%) in 2016 (26) and Salmonella enterica (1.60%) in 2019 (16) in Zhejiang, which suggests that the prevalence of *mcr-1* in *E. fergusonii* is a serious concern. In addition, comparative analysis of *mcr-1*-harboring plasmids and the genetic context of *mcr-1* suggests that there is long-term and complex communication involving *mcr-1* between *E. fergusonii* and other colistin-resistant pathogens. Particularly, the comparative analysis results of *mcr-1*-harboring *E. fergusonii* showed that *E. fergusonii* was prevalent in various hosts, sources, and locations, and that *mcr-1*-negative *E. fergusonii* had the potential ability to carry *mcr-1*. This suggests that the role of *E. fergusonii* in the transmission and development of colistin resistance is more important than previously believed. Therefore, the importance of *E. fergusonii* in the evolution of the *mcr-1* gene should be appreciated, and long-term monitoring of *mcr-1*-positive *E. fergusonii* is necessary.

In summary, the prevalence and characterization of *E. fergusonii* in food animals in China was investigated, as well as its important role in the evolution of antimicrobial resistance. This work suggests that *E. fergusonii* has a high prevalence in food animals and contributes to serious antibiotic resistance. In particular, *E. fergusonii* might facilitate the development of antibiotic resistance as a host of plasmids which harbor resistance genes. Given the potential threat to food safety and public health posed by *E. fergusonii*, long-term monitoring is necessary.

## MATERIALS AND METHODS

### Sample collection and identification of isolates.

A total of 1,400 samples from 410 pigs, 720 chickens, and 270 ducks (feces and anal swabs) were collected between July and September 2019 from 32 different animal farms and slaughterhouses of 14 districts in the Zhejiang, Fujian, and Hunan provinces in China. Detailed information on sample collection is shown in Table S1.

The isolation and identification of *E. fergusonii* was performed based on previously reported methods (27–30). In brief, samples were suspended in buffered peptone water (Land Bridge Technology, Beijing, China) and incubated at 37°C for 18 h. The homogenates were then streaked onto Simmons Citrate Agar plates (Land Bridge Technology, Beijing, China) supplemented with ribitol (5 mL/100 mL; J&K Scientific Ltd., Beijing, China) and incubated at 37°C for 36 to 48 h. The presumptive colony of *E. fergusonii* would be yellow with orange sediment in the center because of fermenting ribitol and d-arabinitol (30, 31). Subsequently, a single typical colony (yellow with orange sediment in the center) on each plate was selected to streak onto a Sorbitol MacConkey Agar plate (Land Bridge Technology, Beijing, China) and incubated at 37°C for 24 h. The presumptive colony would be colorless because it could not ferment sorbitol, while E. coli can ferment sorbitol (32). For each sample, a single presumptive colony (colorless, translucent, and flat) was streaked onto a Luria-Bertani (LB) agar plate (Land Bridge Technology, Beijing, China) and incubated at 37°C for 12 to 18 h. The DNA of presumptive *E. fergusonii* isolates was then prepared using a Bacterial DNA Extraction Kit (Generay Biotech, Shanghai, China). To identify positive *E. fergusonii*, the obtained DNA was used as a template for PCR amplification using the specific primers EF-F (5′-AGATTCACGTAAGCTGTTACCTT-3′) and EF-R (5′-CGTCTGATGAAAGATTTGGGAAG-3′) with 100% identity as previously described (33). E. coli strain ATCC 25922 and a previously isolated *E. fergusonii* strain were used as quality controls.

### Pulsed-field gel electrophoresis and S1-pulsed-field gel electrophoresis.

Pulsed-field gel electrophoresis (PFGE) was performed to determine the genotypic relatedness of *E. fergusonii* isolates, and S1-PFGE was performed to demonstrate the approximate size of plasmids harbored by *E. fergusonii*. The restriction endonucleases *Xba* I and S1 nuclease were used in PFGE analysis and S1-PFGE analysis, respectively. Briefly, after *E. fergusonii* cells were fixed by SeaKem Gold Agarose (Lonza Group AG, USA) and subsequently lysed, the embedded DNAs were digested using *Xba* I or S1 nuclease (Takara Bio, China) in a 37°C water bath for 3 h. The restricted DNA fragments were separated in 0.5× Tris-Borate-EDTA buffer (Sangon Biotech, Shanghai, China) at 14°C for 18 h using a CHEF Mapper electrophoresis system (Bio-Rad, USA) with pulse times of 2.2 to 63.8 s. PFGE images were processed by cluster analysis and band-matching using BioNumerics software (Applied Maths, Sint-Martens-Latem, Belgium) to determine the *E. fergusonii* genotypes and kinships. The band matching was performed with optimization and tolerance settings of 1.0% and 1.5%, respectively. Homology cutoff values of 70% and 85% were used to group the related isolates within the same PFGE-type. Salmonella Braenderup H9812 was used as a standard DNA ladder strain.

### Antimicrobial susceptibility testing.

For all *E. fergusonii* isolates, broth microdilution-based antimicrobial susceptibility testing of Gram-negative pathogens (Biofosun, Fosun Diagnostics, Shanghai, China) was used for testing. A total of 30 antimicrobial agents were used for susceptibility testing (Table S2). The breakpoints for each antimicrobial agent were set by the Clinical and Laboratory Standards Institute (CLSI, 2020: M100-S30) (Table S2). E. coli ATCC 25922, E. coli AR Bank no. 0349, and K. pneumoniae ATCC 700603 served as quality control strains, and the acceptable MIC ranges are shown in Table S2.

The agar dilution method was used to confirm the survival situation of *mcr-1*-harboring *E. fergusonii* strains under colistin pressure (26). The suspensions were prepared from E. coli strain ATCC 25922 and *mcr-1*-harboring *E. fergusonii* strains cultured for 12 to 18 h at 37°C on LB agar. The McFarland standard of the suspensions was adjusted to 0.5 with a densimat (bioMérieux, France). The suspensions were serially diluted 10-fold to 10^−6^, after which 10-μL aliquots of the 10^−2^-to-10^−6^ dilutions for each strain were added to colistin-LB agar at concentrations of 0.25 to 16 μg/mL colistin; LB agar without colistin was used as a negative control. The LB agar with the suspensions was then further cultured at 37°C for 16 h.

### Southern hybridization.

Southern hybridization was performed as described previously (16). The *mcr-1*-specific probe was labeled using a DIG High Prime DNA Labeling and Detection Starter Kit I (Roche, Sant Cugat del Vallés, Spain) following the manufacturer’s instructions.

### Conjugation assay.

Plasmid conjugation experiments were performed on the *mcr-1*-harboring *E. fergusonii* strains as described previously (34). A sodium azide-resistant E. coli strain J53 was used as the recipient. The *mcr-1* specific primers CLR5-F (5′-CGGTCAGTCCGTTTGTTC-3′) and CLR5-R (5′-CTTGGTCGGTCTGTAGGG-3′) were used to confirm the transconjugant as previously reported (13).

### Whole-genome sequencing and sequence analysis.

All *mcr-1*-positive *E. fergusonii* were used to perform whole-genome sequencing. An Illumina sequencing library was generated using a NEXTflex DNA sequencing kit (Bioo Scientific, USA). Genome sequencing was carried out using an Illumina HiSeq X10 platform. Paired-end reads were checked for quality and trimmed with Trimmomatic v0.36. All low-quality (Q < 20) data were filtered out. Finally, raw reads were assembled using SPAdes v3.12.0.

Three strains carrying *mcr-1* plasmids with different sizes were further sequenced to obtain the complete genome sequences. Libraries were prepared by SQK-LSK109 kit (Oxford Nanopore Technologies [ONT], United Kingdom) and sequenced using R9.4 flow cell technology on a GridION sequencer (ONT). All software systems were operated on their default settings. Guppy v3.2.4 was used for base calling of raw fast5 data and removal of adapter sequences. The reads were then assembled using Unicycler. The assembly was corrected by Illumina reads using Pilon v1.22 software and circularized using Circulator v1.5.1. Gene prediction and genome annotation were performed using the NCBI Prokaryotic Genome Annotation Pipeline.

The plasmid replicon types were identified using PlasmidFinder 2.1 (https://cge.cbs.dtu.dk/services/PlasmidFinder/) (35) with a percent identification threshold of 95% and a minimum percent coverage length of 60%. Acquired antimicrobial resistance genes and chromosomal mutations were predicted using ResFinder 4.1 (https://cge.cbs.dtu.dk/services/ResFinder/) (36) (threshold of percent identify = 90%, minimum coverage length = 60%). Easyfig (37) (maximum *E* value = 0.001, identify threshold = 98%) and BRIG (38) (identify threshold = 50%) were used for comparative analysis of the plasmids. Phylogenetic analysis of genome and plasmids was performed by kSNP3 based on the maximum-likelihood method (39). Finally, the phylogenetic tree was generated using MEGA X and iTOL (https://itol.embl.de/).

For convenient sampling, fecal samples were collected for bacterial isolation after the farmer’s verbal consent. All the activities in our study were approved by the institutional review board of the Zhejiang Academy of Agricultural Sciences.

### Data availability.

The complete genomes were deposited in GenBank under the accession numbers JAFIGJ000000000 (EF1), JAFIDW000000000 (EF2), JAFIDX000000000 (EF18), JAFIGK000000000 (EF30), JAFIGO000000000 (EF31), JAFIGP000000000 (EF32), JAFIDO000000000 (EF33), JAFIDQ000000000 (EF43), JAFIDP000000000 (EF44), JAFIDT000000000 (EF45), JAFIGL000000000 (EF51), JAFIGQ000000000 (EF82), JAFIDR000000000 (EF89), JAFIDS000000000 (EF90), JAFIDY000000000 (EF91), JAFIGM000000000 (EF108), JAFIDZ000000000 (EF111), JAFIEA000000000 (EF112), JAFIGN000000000 (EF123), JAFIIL000000000 (EF124), JAFIIM000000000 (EF125), JAFIIN000000000 (EF126), JAFIIO000000000 (EF131), JAFIIP000000000 (EF132), JAFIIQ000000000 (EF133), CP070951 (EF31 chromosome), CP070952 (pTB31P1), CP070953 (pTB31P2), CP070954 (EF44 chromosome), CP070955 (pTB44P1), CP070956 (pTB44P2), CP070957 (pTB44P3), CP070958 (pTB44P4), CP070873 (EF91 chromosome), CP070874 (pTB91P1), and CP070875 (pTB91P2).
