# Supplementary material for: Escherichia fergusonii, an Underrated Repository for Antimicrobial Resistance in Food Animals
Source: Microbiol Spectr. 2022 Feb 9;10(1):e01617-21. doi: 10.1128/spectrum.01617-21 (PMC8826826; doi:10.1128/spectrum.01617-21)
Supplement: SUPPLEMENTAL FILE 1 — Supplemental material. Download SPECTRUM01617-21_Supp_1_seq10.pdf, PDF file, 2.5 MB [file spectrum01617-21_supp_1_seq10.pdf]

Supplemental Figures

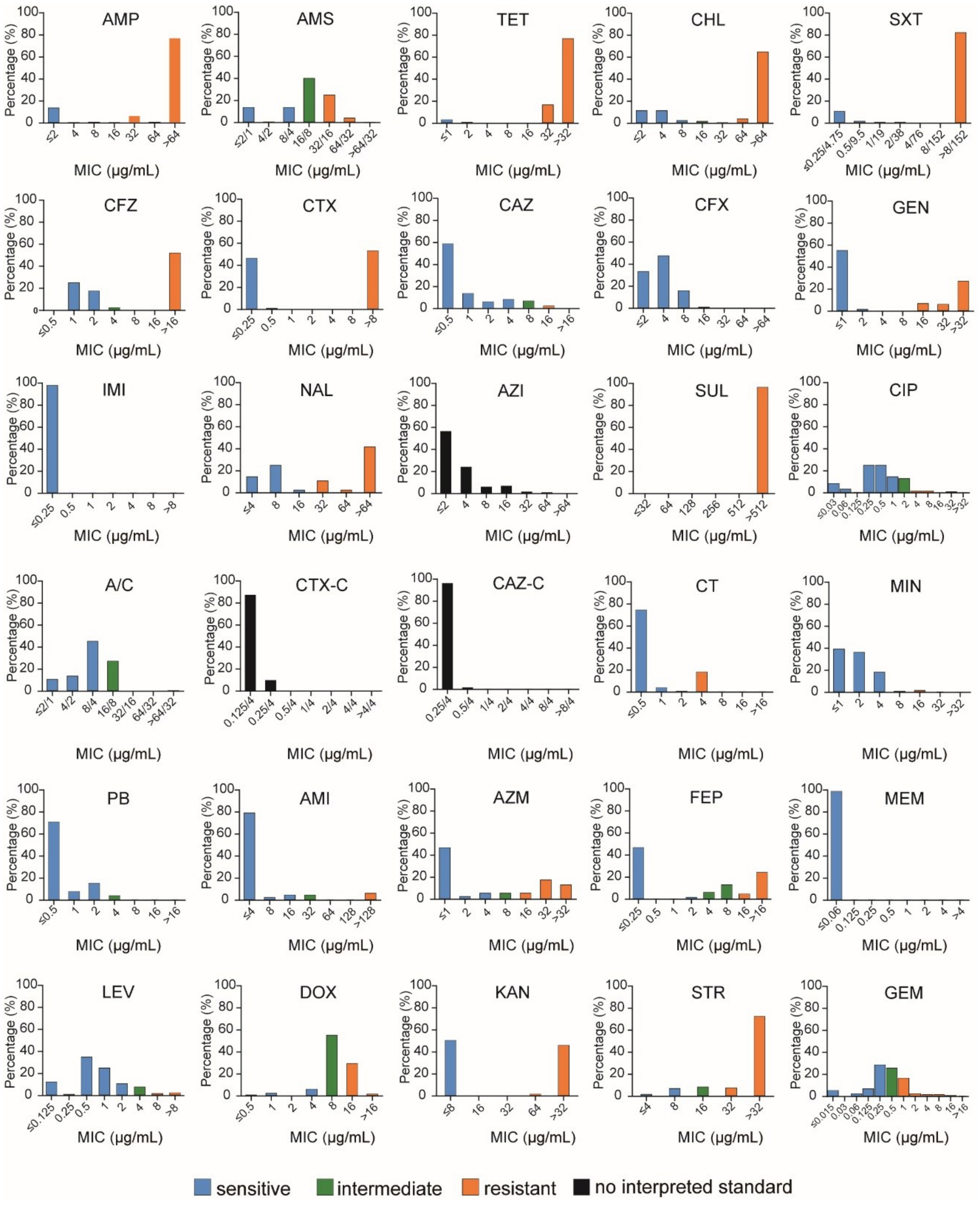

Figure S1. MIC distributions of 133 *E. fergusonii* isolates in this study.

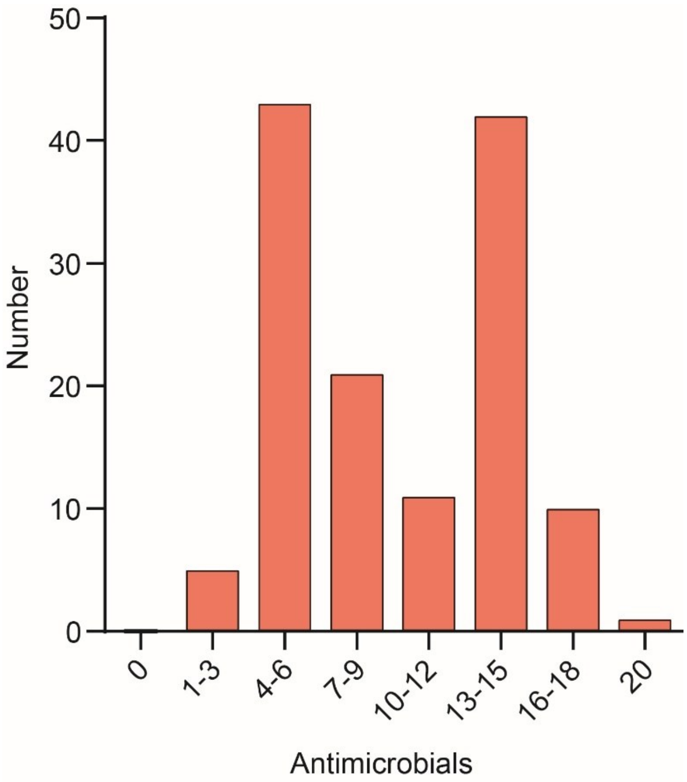

Figure S2. The number of *E. fergusonii* isolates resistant to different numbers of antimicrobials

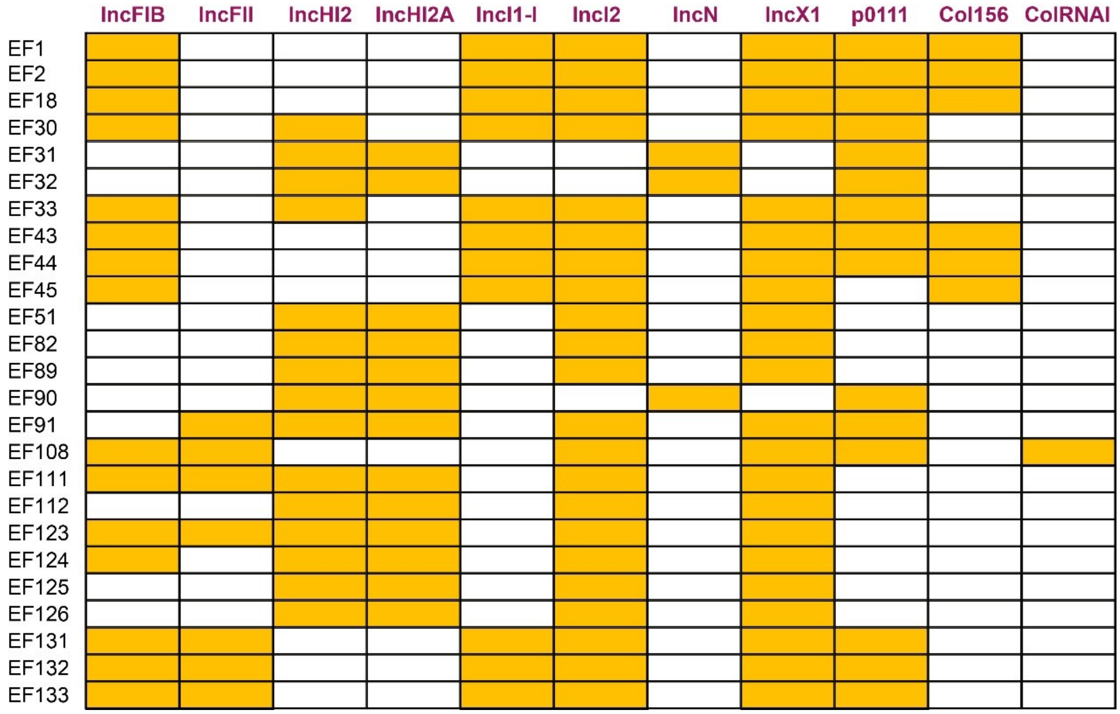

Figure S3. The types of plasmids carried by *mcr-1*-postive *E. fergusonii*

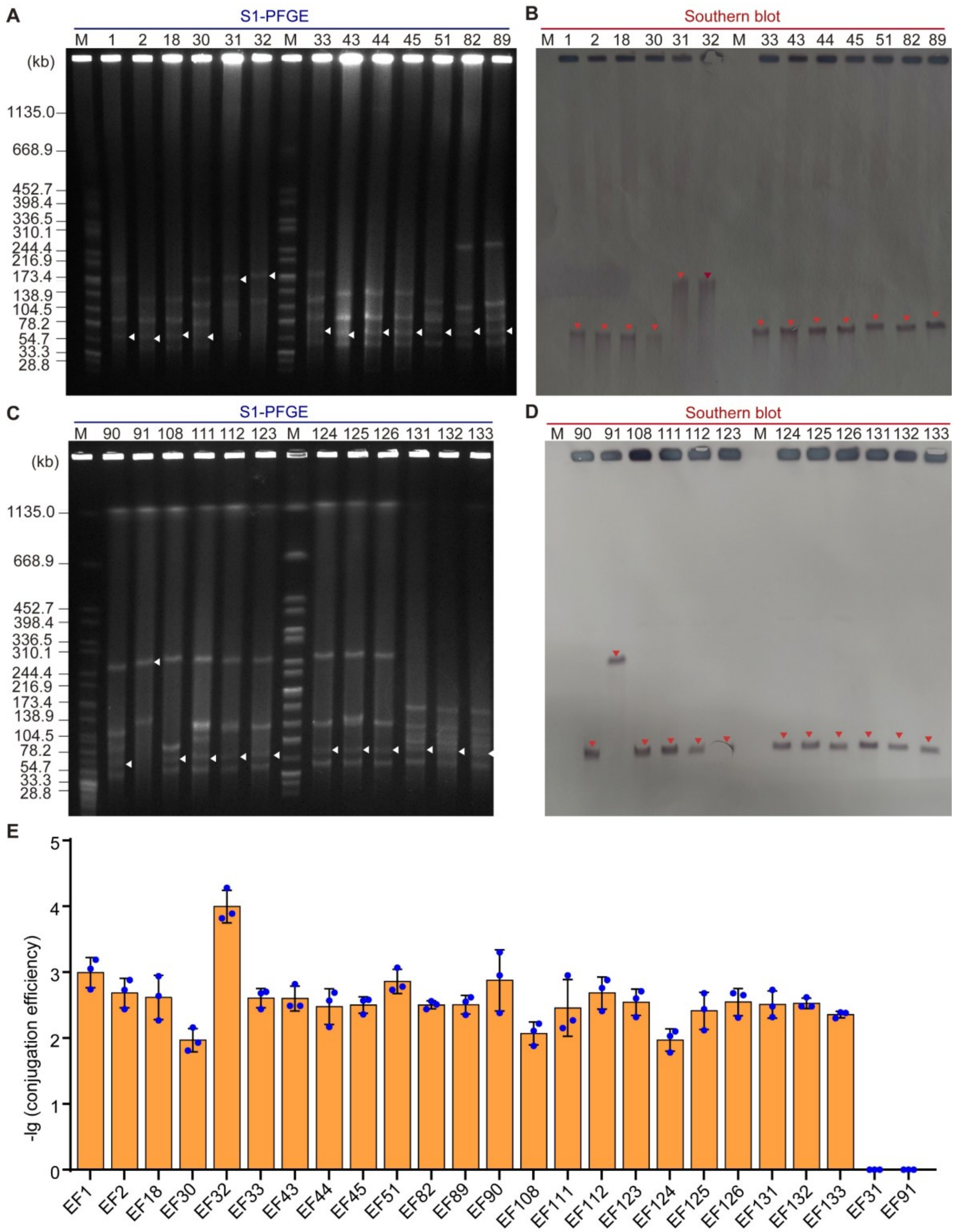

Figure S4. The S1-PFGE, Southern blot and transfer-conjugation test of *mcr-1*-producing *E. fergusonii* isolates. (A) The S1-PFGE of EF1~EF89. (B) The southern blot results of EF1~EF89. (C) The S1-PFGE results of EF90~EF133. (D) The southern blot results of EF90~EF133. (E) Efficiency of conjugation transfer test of *mcr-1*-harboring plasmids in 25 *E. fergusonii* isolates.

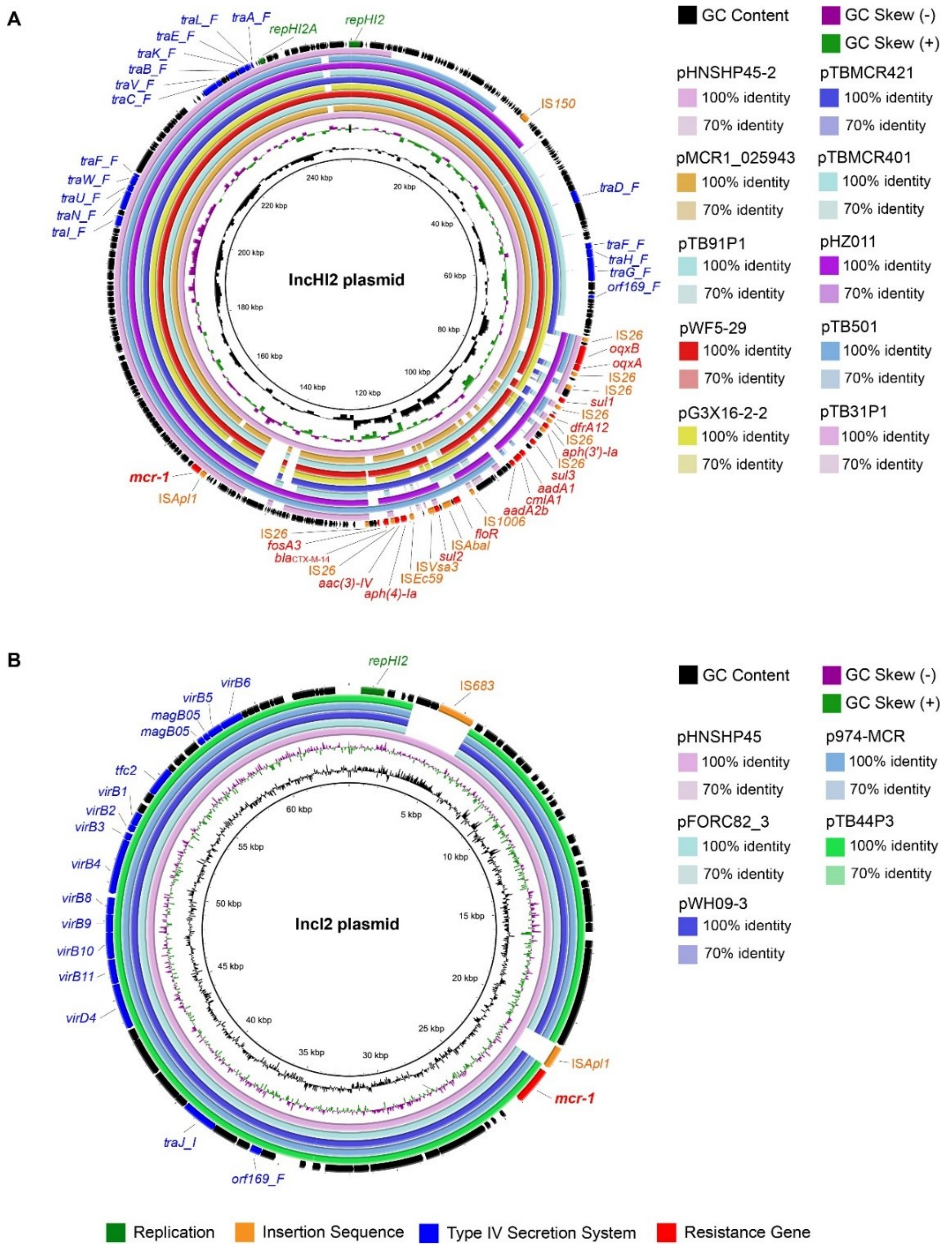

Figure S5. Comparative analysis of pTB31P1, pTB44P3 and pTB91P1 based on the BLAST Ring Image Generator tool. The genes are color coded according to functional annotations. (A) Comparative analysis of pTB31P1 and pTB91P1 with reported IncHI2 plasmids. (B) Comparative analysis of pTB44P3 with reported IncI2 plasmids.

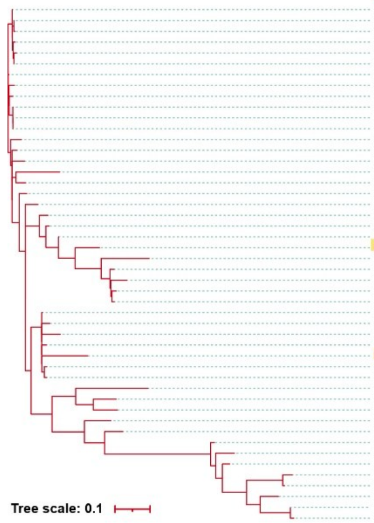

Plasmid name	Strain name	Strain	Accession number	Length (bp)	Host	Source	Location	Collection date
pSH15G1452	SH15G1452	*Salmonella enterica*	MK477607	224296	Homo sapiens	stool	China: Shanghai	2015-10-13
pSH15G1219	SH15G1219	*Salmonella enterica*	MK477603	224296	Homo sapiens	stool	China: Shanghai	2015-9-7
pSH16G0600	SH16G0600	*Salmonella enterica*	MK477612	250732	Homo sapiens	stool	China: Shanghai	2016-5-10
pSH16G4511	SH16G4511	*Salmonella enterica*	MK477618	219141	Homo sapiens	stool	China: Shanghai	2016-7-4
pSH15G1428	SH15G1428	*Salmonella enterica*	MK477605	252852	Homo sapiens	stool	China: Shanghai	2015-9-7
pSH16G4918	SH16G4918	*Salmonella enterica*	MK477619	217630	Homo sapiens	stool	China: Shanghai	2016-8-16
p2474-MCR1	Z247	*Escherichia coli*	CP021209	223982	Homo sapiens	blood	China: Anhui	2015-9-11
pSH16G2456	SH16G2456	*Salmonella enterica*	MK477616	251053	Homo sapiens	stool	China: Shanghai	2016-6-19
pCFSA629	CFSA629	*Salmonella enterica*	CP033351	210688	Food	egg	China: Guangdong	2016-7
pSH16G4926	SH16G4926	*Salmonella enterica*	MK477620	217193	Homo sapiens	stool	China: Shanghai	2016-8-27
pSH15G1169	SH15G1169	*Salmonella enterica*	MK477602	217193	Homo sapiens	stool	China: Shanghai	2015-8-26
pSH16G0612	SH16G0612	*Salmonella enterica*	MK477613	217193	Homo sapiens	stool	China: Shanghai	2016-6-12
pLSB54-mcr-1	LSB54	*Escherichia coli*	MG773376	251657	Unknown	unknown	Unknown	Unknown
pSH15G1450	SH15G1450	*Salmonella enterica*	MK477606	254389	Homo sapiens	stool	China: Shanghai	2015-9-7
pSH16G4466	SH16G4466	*Salmonella enterica*	MK477617	249071	Homo sapiens	stool	China: Shanghai	2016-6-21
p92944-MCR	92944	Escherichia coli	MH329870	198366	Homo sapiens	unknown	Unknown	Unknown
EC1107-IncHI2-226K	EC1107	Escherichia coli	MG662415	226105	Food	chicken	Unknown	2017-3-27
pTB501	SSDFZ54	Salmonella	CP034820	188527	Duck	faeces	China: Zhejiang	2018-3
pSH16G1394	SH16G1394	Salmonella enterica	MK477614	251867	Homo sapiens	stool	China: Shanghai	2016-8-1
pHSHLJ1-MCR1	HSHLJ1	Salmonella enterica	KX856066	238539	Homo sapiens	stool	Unknown	2015
pSH15G2194	SH15G2194	Salmonella enterica	MK477611	263466	Homo sapiens	stool	China: Shanghai	2015-10-28
pXGE1mcr	XG-E1	Escherichia coli	KY990887	254048	Dairy cow	faeces	China: Jiangsu	2015
pTB31P1	EF31	*Escherichia fergusonii*	CP070952	184652	Chicken	feces	China: Zhejiang	2019
p1106-IncHI2	1106	*Escherichia coli*	MG825373	265799	Food	chicken	China	Unknown
pAN65-1	CRE	*Klebsiella pneumoniae*	MK355502	276370	Homo sapiens	faeces	Unknown	Unknown
pEC15-MCR-50	15-50	*Escherichia coli*	MG656414	350179	Unknown	unknown	China	Unknown
pTBMCR421	ECCNB20-2	*Escherichia coli*	CP034788	254365	Food	chicken	China: Zhejiang	2016-6-24
pHN6DS2	GZ6DS2	*Escherichia coli*	MH459020	253783	Dog	urine sample	China: Guangdong	Unknown
pHNSHP45-2	SHP45	*Escherichia coli*	KU341381	251493	Pig	faeces	China: Shanghai	2013-7
pCFSA122-1	CFSA122	*Salmonella enterica*	CP033224	181750	Food	pork dumplings	Unknown	2013-5-1
pECJS-59-244	59	*Escherichia coli*	KX084394	243572	Piglet	faeces	China: Jiangsu	2015-1-19
pASSD2-MCR1	enterica	*Salmonella enterica*	KX856065	187257	Pig	annal swab	Unknown	2015
pTB91P1	EF91	*Escherichia fergusonii*	CP070874	255882	Chicken	annal swab	China: Zhejiang	2019
pSH15G1571	SH15G1571	*Salmonella enterica*	MK477608	175206	Homo sapiens	stool	China: Shanghai	2015-11-14
pSH15G1598	SH15G1598	*Salmonella enterica*	MK477609	176207	Homo sapiens	stool	China: Shanghai	2015-12-28
pECJS-B60-267	JS-B60	*Escherichia coli*	KX254341	267486	Pig	feces	China: Jiangsu	2015-1-19
pSCE516-1		*Escherichia coli*	KX023262	237939	Chicken	unknown	Unknown	Unknown
803-DB-mcr	803DBmcr	*Escherichia coli*	MH128771	230365	Homo sapiens	unknown	China: Zhejiang	2017-3
pCFSA1096	CFSA1096	*Salmonella enterica*	CP033347	297355	Food	pork	China: Hubei	2015-10
pRS571-MCR-1.1	RS571	*Escherichia coli*	CP034390	257270	Homo sapiens	rectal swab	Bangladesh: Dhaka	2018-5-16
pSA26-MCR-1	SA26	*Escherichia coli*	KU743384	240367	Homo sapiens	blood	Saudi Arabia	Unknown
p100R	100R	*Escherichia coli*	KY689633	256260	Homo sapiens	feces	Switzerland	2015
p19M12	19-M12	*Escherichia coli*	KY689632	232345	Homo sapiens	feces	Switzerland	2015
pS38	S38	*Escherichia coli*	KX129782	247885	Food	imported poultry meat	Italy	Unknown
pJMA2	APECA2	*Escherichia coli*	MH208235	224630	Broiler chicken	unknown	Germany	Unknown
pMS8345A	MS8345	*Escherichia coli*	CP025402	241162	Homo sapiens	respiratory secretions	Qatar	2016
pM160133_p1	M160133	*Escherichia coli*	CP022165	233149	Homo sapiens	urine	USA: New York	2016-11-13
pEGY1-MCR-1	CFSAN061770	*Escherichia coli*	CP023143	228947	Food	raw milk cheese	Egypt	2016

Figure S6. Phylogenetic tree of 48 IncHI2-type plasmids harboring *mcr-1* gene with complete sequences. Plasmid sequences were aligned for phylogenetic analysis based on maximum-likelihood.

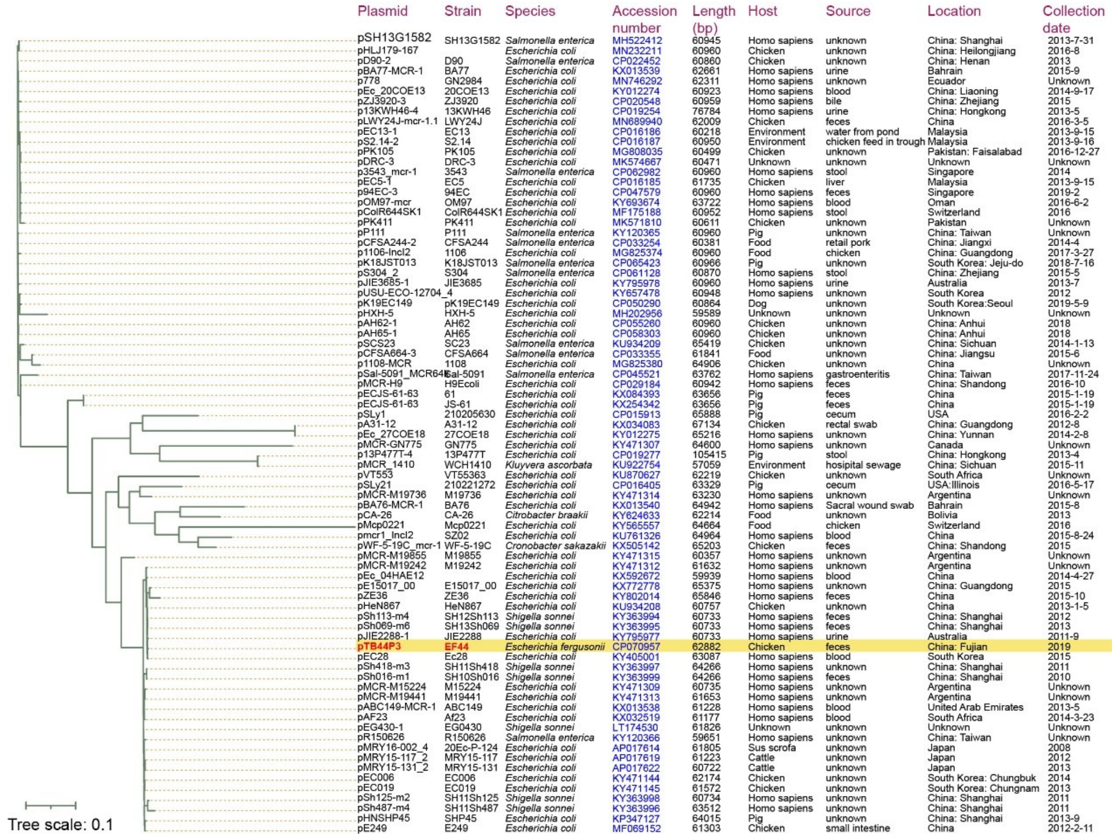

Figure S7. Phylogenetic tree of 79 IncI2-type plasmids harboring *mcr-1* gene with complete sequences. Plasmid sequences were aligned for phylogenetic analysis based on maximum-likelihood.

Table S1. The information of sample collection

No.	Province	City	Location	Date	Type	Number of samples	Total	Number of isolates	Isolation rate (%)
1	Zhejiang	Jiande, Hangzhou	ZCS1 (chicken, slaughter house)	2019.7.20	feces	50	50	4	8
2	Zhejiang	Shaoxing	ZCF2 (chicken, farm)	2019.8.5	feces	30	40	4	10
					anal swab	10			
3	Zhejiang	Shaoxing	ZCS2 (chicken, slaughter house)	2019.8.5	anal swab	50	50	18	36
4	Zhejiang	Ningbo	ZCF6 (chicken, farm)	2019.8.8	feces	40	40	8	20
5	Zhejiang	Ningbo	ZDF3 (duck, farm)	2019.8.8	feces	40	40	4	10
6	Zhejiang	Cixi, Ningbo	ZCF1 (chicken, farm)	2019.8.13	feces	40	40	2	5
7	Zhejiang	Zhuji, Shaoxing	ZDF2 (duck, farm)	2019.8.13	feces	40	40	7	17.5
8	Zhejiang	Jiaxing	ZCF4 (chicken, farm)	2019.8.15	feces	50	50	5	10
9	Zhejiang	Jiaxing	ZCF5 (chicken, farm)	2019.8.15	anal swab	40	40	4	10
10	Zhejiang	Jiaxing	ZPF1 (pig, farm)	2019.8.15	feces	50	50	3	6
11	Zhejiang	Yiwu, Jinhua	ZCF3 (chicken, farm)	2019.8.19	feces	40	40	2	5
12	Zhejiang	Jinhua	ZDF1 (duck, farm)	2019.8.19	feces	50	50	9	18
13	Zhejiang	Jinhua	ZPF2 (pig, farm)	2019.8.19	feces	40	40	4	10
14	Fujian	Fuqing, Fuzhou	FCF2 (chicken, farm)	2019.8.25	feces	40	40	12	30
15	Fujian	Zhangzhou	FCF4 (chicken, farm)	2019.8.26	feces	40	40	1	2.5
16	Fujian	Zhangzhou	FCF5 (chicken, farm)	2019.8.26	feces	40	40	0	0
17	Fujian	Zhangzhou	FDF1 (duck, farm)	2019.8.26	feces	40	40	0	0
18	Fujian	Putian	FCF1 (chicken, farm)	2019.8.27	feces	40	40	3	7.5
19	Fujian	Putian	FPS1 (pig, slaughter house)	2019.8.27	feces	40	40	0	0
20	Fujian	Quanzhou	FCF3 (chicken, farm)	2019.8.28	feces	40	40	0	0

21	Fujian	Xiamen	FPF1 (pig, farm)	2019.8.29	feces	50	50	13	26
22	Fujian	Xiamen	FPF2 (pig, farm)	2019.8.29	feces	40	40	0	0
23	Fujian	Xiamen	FPS2 (pig, slaughter house)	2019.8.29	feces	50	50	8	16
24	Hunan	Changde	HDF1 (duck, farm)	2019.9.13	feces	50	50	0	0
25	Hunan	Changde	HPF1 (pig, farm)	2019.9.13	feces	50	50	0	0
26	Hunan	Zhuzhou	HCF1 (chicken, farm)	2019.9.15	anal swab	40	40	6	15
27	Hunan	Zhuzhou	HCF2 (chicken, farm)	2019.9.15	feces	40	40	5	12.5
28	Hunan	Zhuzhou	HCF3 (chicken, farm)	2019.9.15	feces	40	40	7	17.5
29	Hunan	Zhuzhou	HPF2 (pig, farm)	2019.9.15	feces	40	40	1	2.5
30	Hunan	Ningxiang, Changsha	HDF2 (duck, farm)	2019.9.17	feces	50	50	0	0
31	Hunan	Binzhou	HCF4 (chicken, farm)	2019.9.18	feces	50	50	3	6
32	Hunan	Binzhou	HPF3 (pig, farm)	2019.9.18	feces	50	50	0	0
	In total						1400	133	9.5

Table S2. The antibiotics, breakpoints and quality control strains used in antimicrobial susceptibility testing for *E. fergusonii* isolates

Antibiotic	Breakpoint (μg/mL)	Reference	Quality Control *E. coli* ATCC 25922 (μg/mL)	Quality Control *E. coli* AR Bank #0349 (μg/mL)	Quality Control *K. pneumoniae* ATCC 700603 (μg/mL)
**Beta-Lactamase**					
Ampicillin (AMP)	≥32	CLSI, 2020: M100-S30	2-8		
Ceftazidime (CAZ)	≥16	CLSI, 2020: M100-S30	0.06-0.5		
Cefazolin (CFZ)	≥8	CLSI, 2020: M100-S30	1-4		
Cefotaxime (CTX)	≥4	CLSI, 2020: M100-S30	0.03-0.12		8
Cefoxitin (CFX)	≥32	CLSI, 2020: M100-S30	2-8		32
Cefepime (FEP)	≥16	CLSI, 2020: M100-S30	0.016-0.12		
Amoxicillin/clavulanic acid (A/C)	≥32/16	CLSI, 2020: M100-S30	2/1-8/4		
Ampicillin/Sulbactam (AMS)	≥32/16	CLSI, 2020: M100-S30	2/1-8/4		
Cefotaxime/clavulanic acid (CTX-C)[a]	a	CLSI, 2020: M100-S30	-		≤0.25
Ceftazidime/clavulanic acid (CAZ-C)[a]	a	CLSI, 2020: M100-S30	-		1
Aztreonam (AZM)	≥16	CLSI, 2020: M100-S30	0.06-0.25		
Meropenem (MEM)	≥4	CLSI, 2020: M100-S30	0.008-0.06		
Imipenem (IMI)	≥4	CLSI, 2020: M100-S30	0.06-0.25		
**Aminoglycoside**					
Gentamicin (GEN)	≥16	CLSI, 2020: M100-S30	0.25-1		
Amikacin (AMI)	≥64	CLSI, 2020: M100-S30	0.5-4		
Kanamycin (KAN)	≥64	CLSI, 2020: M100-S30	1-4		
Streptomycin (STR)	≥32	CLSI, 2020: M100-S30	-		
**Tetracycline**					
Tetracycline (TET)	≥16	CLSI, 2020: M100-S30	0.5-2		
Minocycline (MIN)	≥16	CLSI, 2020: M100-S30	0.25-1		
Doxycycline (DOX)	≥16	CLSI, 2020: M100-S30	0.5-2		
**Chloramphenicol**					

Chloromycetin (CHL)	≥32	CLSI, 2020: M100-S30	2-8	
**Sulphonamide**				
Sulfafurazole (Sul)	≥512	CLSI, 2020: M100-S30	8-32	
Sulfamethoxazole (SXT)	≥4/76	CLSI, 2020: M100-S30	≤0.5/9.5	
**Quinolone**				
Nalidixic acid (NAL)	≥32	CLSI, 2020: M100-S30	1-4	
Ciprofloxacin (CIP)	≥4	CLSI, 2020: M100-S30	0.004-0.015	
Levofloxacin (LEV)	≥8	CLSI, 2020: M100-S30	0.008-0.06	
Gemifloxacin (GEM)[b]			0.004-0.016	
**Macrolides**				
Azithromycin (AZI)[b]			-	
**Peptide**				
Colistin (CT)	≥4	CLSI, 2020: M100-S30	0.25-2	4
Polymyxin B (PB)	≥8	CLSI, 2020: M100-S30	0.25-2	

[a] ≥3 twofold concentration decrease in an MIC for either antimicrobial agent tested in combination with clavulanate *vs* the MIC of the agent when tested alone = ESBL (eg, ceftazidime MIC = 8 μg/mL; ceftazidime-clavulanate MIC = 1 μg/mL).

[b] No interpreted standard for this antibiotic.

Table S4. Chromosomal point mutations in 25 *mcr-1*-harboring *E. fergusonii* isolates

Strain	Chromosomal point mutation	Antibiotic-resistant phenotype
EF1	*gyrA*:p.S83L	Quinolone
EF2	*gyrA*:p.S83L	Quinolone
EF18	*gyrA*:p.S83L	Quinolone
EF30	*gyrA*:p.S83L	Quinolone
EF31	-	-
EF32	-	-
EF33	*gyrA*:p.S83L	Quinolone
EF43	*gyrA*:p.S83L	Quinolone
EF44	*gyrA*:p.S83L	Quinolone
EF45	*gyrA*:p.S83L	Quinolone
EF51	*gyrA*:p.S83L	Quinolone
EF82	*gyrA*:p.S83L	Quinolone
EF89	*gyrA*:p.S83L	Quinolone
EF90	-	-
EF91	*gyrA*:p.S83L	Quinolone
EF108	*gyrA*:p.S83L	Quinolone
EF111	*gyrA*:p.S83L	Quinolone
EF112	*gyrA*:p.S83L	Quinolone
EF123	*gyrA*:p.S83L	Quinolone
EF124	*gyrA*:p.S83L	Quinolone
EF125	*gyrA*:p.S83L	Quinolone
EF126	*gyrA*:p.S83L	Quinolone
EF131	*gyrA*:p.S83L	Quinolone
EF132	*gyrA*:p.S83L	Quinolone
EF133	*gyrA*:p.S83L	Quinolone
